# Health-related quality of life (EQ-5D) before and after orthopedic surgery

**DOI:** 10.3109/17453674.2010.548026

**Published:** 2011-02-10

**Authors:** Karl-Åke Jansson, Fredrik Granath

**Affiliations:** ^1^Orthopedics Section, Department of Molecular Medicine and Surgery, Karolinska Institutet, at Karolinska University Hospital; ^2^Clinical Epidemiology Unit, Department of Medicine, Karolinska Institutet, at Karolinska University Hospital, Stockholm, Sweden

## Abstract

**Background and purpose:**

Population data on mortality and life expectancy are generally available for most countries. However, no longitudinal data based on the health-related quality of life outcome from the EQ-5D instrument have been reported for orthopedic patients. We assessed the effect of orthopedic surgery as measured by EQ-5D.

**Methods:**

We analyzed EQ-5D data from 2,444 patients who were operated at the Department of Orthopedic Surgery at Karolinska University Hospital, 2001–2005. We also made a comparison between results from this cohort and those from a Swedish EQ-5D population survey.

**Results:**

The mean EQ-5D _index_ score improved from 0.54 to 0.72. Hip and knee arthroplasty, operations related to previous surgery, trauma-related procedures, and rheumatoid arthritis surgeries had preoperative EQ-5D _index_ scores of 0.48 to 0.52. All of these groups showed substantial improvement in scores (0.63 to 0.80). Patients with tumors or diseases of the elbow/hand showed higher preoperative scores (0.66 to 0.77), which were similar postoperatively. In most patients, the EQ-5D _index_ score improved but did not reach the level reported for an age- and sex-matched population sample (mean difference = 0.11).

**Interpretation:**

Our results can be used as part of the preoperative patient information to increase the level of patient awareness and cooperation, and to facilitate rehabilitation. In future it will be possible—but not easy—to use the EQ-5D instrument as a complementary consideration in clinical priority assessment.

Musculoskeletal conditions are the leading cause of severe long-term pain and disability in the world, affecting hundreds of millions of people (Woofle and Pfleger 2003). They are also the main cause of disability in older age groups, and rank among the top 10 causes of disability-adjusted life-years (DALY) in Europe (WHO 2006). This has been recognized by the World Health Organization, endorsing the Bone and Joint Decade (2000–2010) (Woolfe 2000). Osteoarthritis is the fifth greatest cause of years lived with disability (YLD) in high-income countries (The Word Bank 2006). During the year 2007, 114,000 patients underwent a primary hip or knee joint replacement operation in the UK (England and Wales National Joint Registry 2009). Prevalence data from Sweden for the same year show that 1 in 15 elderly women had a knee arthroplasty (Swedish Knee Arthroplasty Register 2009). One of the major goals of the Bone and Joint Decade has been to reduce the burden and cost of musculoskeletal disorders for individuals, healthcare providers, and society in general. At the end of the decade, it is now appropriate to reflect on the outcome of orthopedic surgery.

Improvement in health-related quality of life (HRQOL) is one of the most important goals of orthopedic surgery (Ethgen et al. 2006, Jansson et al. 2009). There are several HRQOL instruments available. Among these, the generic instruments can be used for diverse patient groups independently of the underlying disease or disability. Generic instruments include, for example, the EQ-5D (EuroQol), the SF-6D (derived from RAND-36/SF-36), the HUI (Health Utilities Index Mark II/Mark III), and the AQoL (Assessment of Quality of Life) (Kopec and Willison 2003). The SF-36 instrument is most commonly used. Most studies have concentrated on specific orthopedic interventions, and most of them show improved HRQOL after surgery (Towheed and Hochberg 1996). HRQOL has been used to evaluate the effect of surgical procedures (Hoffmann et al. 2006, Akahane et al. 2007). Treatment outcome across various elective orthopedic surgical procedures has been compared (Hansson et al. 2008, Anderson et al. 2009, Osnes-Ringen et al. 2009). Generic tools have also been used for the estimation of orthopedic effectiveness of healthcare (Räsänen et al. 2006). The generic health-related quality of life instrument—EQ-5D—allows both a description of health status along 5 dimensions and the evaluation of health or the estimation of a health summary score: the EQ-5D score on a scale where 0 is death and 1 is full health (Dolan 1997, Brooks et al. 2003). The instrument has been included in population surveys in more than 10 countries (Kind et al. 1998, Burström et al. 2001, Scende and Williams 2004). HRQOL and health status measures have often been used as outcomes in clinical trials and studies assessing a variety of orthopedic interventions (Tidermark et al. 2003, Cockerill et al. 2004, Jansson et al. 2005, Löfvendahl et al. 2005, Rivero-Arias et al. 2005, Odenbring et al. 2008, Giannoudis et al. 2009).

The EQ-5D is short and easy to use, and shows good responsiveness (Tidermark et al. 2003), i.e. it is capable of capturing clinically important changes. Moreover, it also allows combination of different dimensions of health to form an overall index, the EQ-5D _index_ score, as required for healthcare evaluations and for construction of quality-adjusted life-years (QALYs), a measure frequently used in cost-effectiveness analyses (Gold et al. 1996, Meunning and Gold 2001, Drummond et al. 2005).

Population data on mortality and life expectancy are generally available for most countries. However, no longitudinal data based on the inclusion of the HRQOL outcome by the EQ-5D have been reported in a clinical setting of orthopedic patients. We therefore introduced the EQ-5D instrument at our department in order to measure all patients selected for elective orthopedic operations. The aim of this study was to preoperatively evaluate the HRQOL in our cohort regardless of other co-morbidity factors and also to make a comparison between this cohort and a Swedish EQ-5D population survey. In addition, we wanted to assess the postoperative outcome by the EQ-5D instrument in order to have output data to explore the potential of EQ-5D for medical priority and health economy calculations. We report data from 2,444 patients.

## Patients and methods

### Study population

Between January 2001 and May 2005, 4,715 elective orthopedic operations were performed at the Department of Orthopedic Surgery, Karolinska University Hospital. We included 4,011 patients during this period, all of whom completed the EQ-5D questionnaire. Acute operations were not included.

The enrollment of patients was done at the ward, and informed consent was given by all patients. At baseline, i.e. on the day before surgery, the first EQ-5D questionnaire was completed by the patient at the ward. The 12-month EQ-5D questionnaire was sent once to the patients by mail 11 months after surgery once, with no reminders. To be included in the 12-month follow-up, patients had to have answered the EQ-5D questionnaire within 3 months. 2,444 patients completed the 12-month EQ-5D questionnaire within 15 months postoperatively. We performed a drop-out analysis of the 1,567 patients who did not answer the 12-month follow-up questionnaire. Age, sex, type of surgery, and preoperative EQ-5D data were scrutinized.

We divided the cohort into 15 groups according to anatomical region and type of surgery. We also compared EQ-5D results for patients older than 20 years of age with those from a Swedish population survey involving 3,069 individuals (Burström et al. 2001, 2003).

The study design was approved by the ethics committee of Karolinska Institutet (no. 03-631).

### Outcomes: the EQ-5D measure

Health-related quality of life data were obtained from the EQ-5D, a self-administered patient questionnaire (EuroQol Group 1990, Brooks 1996, Dolan et al. 1996). The EQ-5D respondents classify their own health status into 5 dimensions: mobility, self-care, usual activities, pain/discomfort, and anxiety/depression with 3 levels of severity (no problems, moderate problems, or severe problems). Dolan et al. (1996) used the time trade-off (TTO) method to rate these different states of health in a large UK population (UK EQ-5D _index_ tariff). As there is no Swedish TTO tariff for EQ-5D health states, and since the only Swedish population survey to assess the EQ-5D used the UK tariff, we used the preference scores generated from the UK population when calculating the EQ-5D _index_ scores for our study population. The patients completed the Swedish-translated questionnaire (EQ-5D 2009). By design, this descriptive system is able to identify 243 unique health states. An index score can be assigned to each of these health states to indicate its value or desirability from the point of view of the general public. Scores in the UK EQ-5D value set range from –0.594 for the worst possible health state to 1.0 for a perfect state of health, with 0 on the scale representing the state of being dead. Negative scores suggest that the corresponding health states are considered worse than being dead. Normally, the EQ-5D questionnaire needs 1 to 3 min for self-completion.

### Statistics

The EQ-5D _index_ scores are reported as mean (SD). Age and sex standardized EQ-5D index scores at baseline (preoperatively and at 12 month follow-up (postoperatively) was calculated as the difference between observed scores and the age- (10 year age-groups) and sex-specific mean scores in the population survey. These preoperative and (12-month) postoperative EQ-5D _index_ standardized scores are reported as mean (SD).

The changes in EQ-5D _index_ score from baseline (preoperatively) and 12 months (postoperatively) were calculated and a paired t-test was used to test whether the change from baseline was equal to 0. We also analyzed the fraction of patients (by number and percentage) whose EQ-5D _index_ score changed from baseline to 12 months (improved or deterioriated by > 0.1). Responders and non-responders at the 12-month follow-up were compared regarding age, sex, type of surgery, and preoperative EQ-5D by chi-square tests for qualitative variables and t-tests for quantitative variables. Even though the distribution of the change from baseline was not normal, the central limit theorem implies valid inference using the t-test when the sample size is more than about 30, and all but 1 subgroup had larger sample sizes. Since the fraction of responders was different for the different types of surgery, the comparisons with respect to age, sex, and preoperative EQ-5D score were also adjusted for this difference by analysis of variance and logistic regression.

## Results

The final analysis included 2,444 patients, 57% of whom were women, and the mean age at surgery was 56 (SD 18) years (Table 1). One third of the patients had osteoarthritis and were operated on for hip or knee replacement. 13% of the patients had operations due to complications after previous surgery. 1 in 10 had trauma related to surgery, and 1 in 10 was operated due to knee disorders. 1.4% had an unknown, unidentified operation procedure code.

**Table 1.** Details of the 2,444 patients in the study at baseline (preoperatively), including surgical procedures and anatomical regions

			Age		Females	EQ-5D index score at baseline		Standardized score **^a^** at baseline	
	N	%	mean	SD	%	mean	SD	mean	SD
All patients	2,444	100	56	18	56	0.54	0.35	–0.29	0.35
Sex									
Women	1,359	55.6	59	18	–	0.50	0.37	–0.36	0.36
Men	1,085	44.4	50	18	–	0.59	0.33	–0.26	0.33
Op. procedure									
Hip arthroplasty	370	15.1	69	11	55	0.49	0.34	–0.31	0.34
Knee arthroplasty	365	14.9	67	12	61	0.51	0.33	–0.29	0.34
Complications	326	13.3	53	19	52	0.52	0.37	–0.31	0.36
Trauma	287	11.7	46	19	48	0.52	0.36	–0.34	0.35
Knee	210	8.6	43	17	45	0.65	0.30	–0.21	0.30
Benign tumor	173	7.1	43	17	58	0.77	0.28	–0.09	0.27
Rheumatoid arthritis	159	6.5	59	13	84	0.48	0.36	–0.34	0.36
Malignant tumor	119	4.9	58	19	53	0.71	0.31	–0.11	0.31
Spine	119	4.9	58	16	54	0.30	0.35	–0.53	0.35
Hip	95	3.9	54	18	60	0.41	0.36	–0.43	0.35
Shoulder	74	3.0	51	16	43	0.62	0.36	–0.23	0.35
Foot	51	2.1	50	15	63	0.56	0.35	–0.28	0.36
Elbow/hand	37	1.5	55	14	59	0.67	0.29	–0.16	0.29
Diabetes/infections	26	1.1	62	17	42	0.40	0.37	–0.41	0.38
Unknown	33	1.4	45	17	30	0.58	0.35	–0.28	0.35

**^a^** Standardized EQ-5D score: difference between the preoperative EQ-5D _index_ score (baseline) and that of the reference population survey (age- and sex-specific mean EQ-5D _index_ scores).

The mean preoperative EQ-5D _index_ score at baseline was 0.54, which is 0.29 units lower than would be expected in a population-based sample of the same age and sex distribution. On average, women had lower scores (0.50) than men (0.59) before surgery (p < 0.001), which remained unchanged after adjustment for age and type of surgery. Age did not affect the preoperative score substantially, with the exception of patients younger than 30 years, who had a higher mean score (by 0.12 units) than the average patients. This age effect could to some extent be explained by type of surgery. The different surgical procedures showed a wide spectrum of average EQ-5D _index_ scores at baseline (0.30–0.77).

When comparing the different surgical procedures for the overall mean EQ-5D _index_ score at baseline, procedures related to benign or malignant tumors and elbow/hand diseases scored statistically significantly higher than average, which is important clinically, while patients with hip and spine procedures scored significantly lower than average.

Preoperatively, at baseline, the standardized EQ-5D _index_ score (mean difference between the age- and sex-matched population) was –0.29. All 15 groups of patients had a lower EQ-5D _index_ score than in the matched population (–0.09 to –0.53).

At the 12-month follow-up, the mean EQ-5D _index_ score had increased statistically significantly by 0.18 units from baseline to 0.72 (Table 2). The mean EQ-5D in women increased almost to the level of that in men: 0.71 in comparison to 0.73. Patients younger than 30 years had a 12-month mean EQ-5D _index_ score of 0.79 and patients older than 80 years had a 12-month mean score of 0.66.

**Table 2.** Details of the 2,444 patients in the study at 12 months postoperatively

			EQ-5D index score 12-month		Standardized score ^a^ 12-month		Change from baseline		Change from baseline > 0.1		Change from baseline < –0.1	
	N	%	mean	SD	mean	SD	mean	p-value ^b^	%	n	%	n
All patients	2,444	100	0.72	0.30	–0.11	0.30	0.18	<0.0001	49	1193	14	334
Sex												
Women	1,359	56	0.71	0.30	–11	0.30	0.21	<0.0001	50	680	12	163
Men	1,085	44	0.73	0.30	–12	0.31	0.14	<0.0001	47	510	16	174
Op. procedure												
Hip arthroplasty	370	15	0.80	0.25	0.00	0.25	0.31	<0.0001	69	254	6	22
Knee arthroplasty	365	15	0.73	0.27	–0.07	0.28	0.22	<0.0001	54	196	9	34
Complications	326	13	0.63	0.34	–0.20	0.34	0.11	<0.0001	40	132	18	60
Trauma	287	12	0.73	0.29	–0.12	0.28	0.21	<0.0001	56	162	12	35
Knee	210	8.6	0.73	0.29	–0.13	0.29	0.09	<0.0001	35	74	13	27
Benign tumor	173	7.1	0.80	0.28	–0.06	0.28	0.03	0.09	32	56	18	31
Rheumatoid arthritis	159	6.5	0.64	0.31	–0.18	0.31	0.16	<0.0001	48	76	11	17
Malignant tumor	119	4.9	0.71	0.28	–0.11	0.28	–0.00	0.97	24	28	31	37
Spine	119	4.9	0.61	0.35	–0.21	0.35	0.31	<0.0001	56	67	14	17
Hip	95	3.9	0.68	0.34	–0.15	0.33	0.27	<0.0001	58	55	13	12
Shoulder	74	3.0	0.73	0.32	–0.12	0.33	0.11	0.005	46	34	19	14
Foot	51	2.1	0.69	0.28	–0.15	0.29	0.13	0.02	41	21	18	9
Elbow/hand	37	1.5	0.70	0.27	–0.13	0.27	0.03	0.57	24	9	24	9
Diabetes/infections	26	1.1	0.66	0.30	–0.15	0.28	0.27	0.002	62	16	8	2
Unknown	33	1.4	0.69	0.32	–0.17	0.32	0.11	0.06	39	13	24	8

^a^ Standardized EQ-5D score: difference between the postoperative EQ-5D _index_ score and that of the reference population survey (age- and sex-specific mean EQ-5D _index_ score).

^b^ p-value for testing if the change from baseline is equal to 0.

Patients who underwent hip or knee arthroplasty, had complications after surgery, underwent other knee surgery, had trauma-related procedures, had rheumatoid arthritis or who underwent spine, hip, or infection-related surgery showed statistically significant improvements in mean EQ-5D _index_ score (0.09 to 0.31). Patients with benign or malignant tumors or elbow/hand diseases showed no statistically significant changes in EQ-5D _index_ score.

The standardized EQ-5D _index_ score (mean difference between the age- and gender-matched population) at 12 months of follow-up was –0.11. Hip arthroplasty patients had a mean standardized EQ-5D _index_ score preoperatively of –0.31 but their EQ-5D score improved and reached the level of that of the age- and sex-matched population (standardized EQ-5D _index_ score of 0.00 at 12-month follow-up). Knee arthroplasty, trauma-related operations, other hip and knee surgery, rheumatoid arthritis surgery, surgery after complications, and spine surgery showed major improvements in EQ-5D _index_ score 12 months after operation. However, they did not reach that of the matched population. The mean difference in score from that of the matched population postoperatively varied from –0.07 to –0.21.

One year after surgery, half of the patients experienced an improvement of > 0.1 in their EQ-5D _index_ score and a small group (14 %) reported deterioration in their scores of > –0.1. 69% of the hip arthroplasty patients improved by at least 0.1 and only 6% deteriorated in their EQ-5D _index_ score, in contrast to malignant tumor surgery where only 24% improved more than 0.1 and 30% deterioriated by > –0.1.

**Figure 1.**
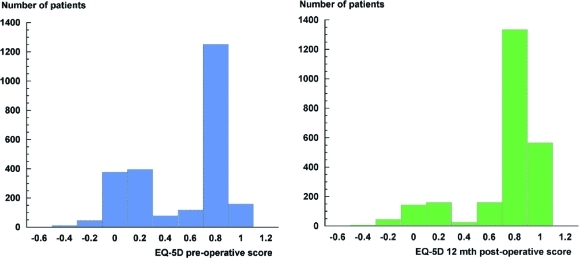
A. Bar chart showing preoperative health-related quality of life (EQ-5D) in the orthopedic cohort. Baseline EQ-5D _index_ scores; n = 2,444. B. Bar chart showing postoperative health-related quality of life (EQ-5D) in the orthopedic cohort. 12-month follow-up EQ-5D _index_ scores; n = 2,444.

**Figure 2.**
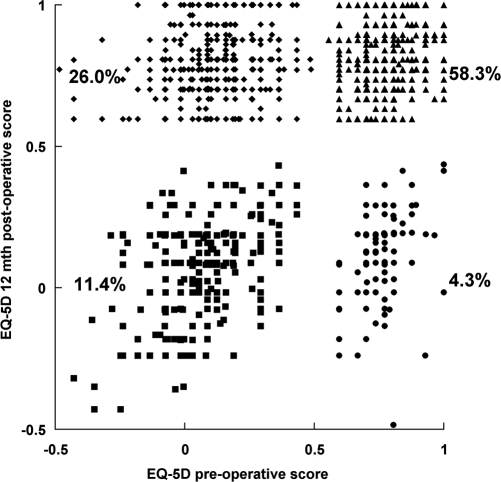
Graph showing health-related quality of life (EQ-5D) in an orthopedic cohort. Preoperative and 12-month postoperative EQ-5D _index_ scores. The first group of patients (26%) had experienced a great improvement (diamonds); a second group of patients (58.3%) with high preoperative scores were slightly improved (triangles). A third group (11.4%) were unchanged, with low EQ-5D _index_ scores (squares), and a fourth, small group (4.3%) had a decline in their scores (circles).

We found that the distribution of the EQ-5D _index_ score was bimodal, and very few individuals scored around the average (Figure 1A and B). Preoperatively, the EQ-5D _index_ score had a bimodal distribution around 0.1 and 0.7. At 12 months, the distribution was still bimodal but most patients now had scores within the range 0.7–1.0. The pre-and postoperative EQ-5D _index_ scores showed 4 major groups of patients (Figure 2). The first group of patients (26%) had experienced great improvement, while a second group of patients with high preoperative scores (58%) had improved slightly. A third group with low EQ-5D scores preoperatively (12%) were unchanged, and a fourth small group (4%) perceived a decline in their HRQOL.

The mean response rate of those who completed the EQ-5D questionnaire at baseline was 85% (Table 3). The response rate varied considerably (59–100%), with the lowest response rates for patients with diabetes/infection (59%) and malignant tumors (68%).

**Table 3.** Total number of elective operations in the study at baseline and responder rate at baseline

	Elective operations at baseline N	Responders at baseline N	%
All	4,715	4,011	85
Op. procedure			
Hip arthroplasty	754	533	71
Knee arthroplasty	612	583	95
Complications	542	524	97
Trauma	576	514	89
Knee	383	383	100
Benign tumor	398	343	86
Reumatoid arthritis	260	220	85
Malignant tumor	317	217	68
Spine	239	186	78
Hip	194	144	74
Shoulder	176	143	81
Foot	85	76	89
Elbow/hand	59	57	97
Diabetes/infections	64	38	59
Unknown	56	50	89

In the dropout analyses (Appendix) we found that the responders were more likely to be women, to be older, or to have a low preoperative EQ-5D _index_ score. The response rate also depended on the type of surgery. The responders were on average 5 years older than non-responders (p < 0.001). However, after adjustment for type of surgery this difference was reduced to 3 years, but it was still highly significant (p < 0.001). A comparison between gender and response rate showed that women had a higher response rate (unadjusted comparison, p = 0.0009). Adjustment of the association between gender and response rate for type of surgery reduced the association between gender and response rate (adjusted, p = 0.02). Similarly, after adjusting the difference in mean EQ-5D _index_ score at baseline between responders and non-responders for type of surgery, the difference became less pronounced. On average, the responders had a lower score than non-responders by 0.05 units (p < 0.001). However, after adjustment for type of surgery, this difference was reduced to 0.03 units (p = 0.02).

## Discussion

We found that most patients who were operated on for orthopedic conditions experienced an improved health-related quality of life and that their mean EQ-5D _index_ score increased from 0.54 to 0.72 one year after surgery.

As expected, we noted large differences between surgical groups. In contrast to patients with tumor diseases, who scored high with a mean EQ-5D of 0.71, patients scheduled for hip or knee arthroplasty scored considerably lower (0.49 and 0.51, respectively). The indication for surgery is however, totally different in these cases, which must be kept in mind when interpreting these data. Notably, the group of patients treated with hip arthroplasty improved considerably and reached the scores of the age- and sex-matched reference population. Interestingly, patients with tumors improved in HRQOL to some extent in spite of their malignant conditions.

In a review evaluating changes after hip replacement, the results from all studies were consistent in showing beneficial and often dramatic improvements in HRQOL after elective procedures (Towheed and Hochberg 1996). Another review analyzing Short Form-36 and the Western Ontario and McMaster University osteoarthritis index after hip and knee arthroplasties showed similar results, and both procedures were found to be quite effective in terms of improvement in health-related quality-of-life dimensions (Ethgen et al. 2004). Surgery for lumbar spinal stenosis can give improvement in self-reported quality of life similar to that in hip and knee arthroplasty (Rampersaud 2008). A recently published study demonstrated that spinal surgery can return patients' HRQL to that of age-matched population norms and yield outcomes similar to those in hip and knee replacement patients (Mokhtar et al. 2010). As other authors have shown (Hansson et al. 2008, Anderson et al. 2009), our study confirms that patients who have undergone spine procedures improve in HRQOL as excellently as the arthroplasty patients do.

In a study evaluating patients with inflammatory arthritis using both EQ-5D and SF-6D health assessment questionnaires, the authors recommended the inclusion of at least one preference-based measure in future clinical studies (Harrison et al. 2010). We noticed in our study that inflammatory arthritis (rheumatoid arthritis (RA)) patients had a positive effect on HRCOL but the improvement was less than for patients treated with joint replacement. The reason for this could be that surgery had an effect on pain in the actual joint treated but less improvement in other dimensions of health (Osnes-Ringen 2009).

The minimal important difference (MID) is important for interpreting the impact of score changes, and is also an important measure for power calculations in studies (Walters and Brazier 2003). MID for EQ-5D _index_ score has been reported by Walters and Brazier (2005). For those subjects who reported some changes, a mean EQ-5D _index_ score of 0.07 was found. In our orthopedic cohort, half of the patients had elevated EQ-5D _index_ scores (by more than 0.1) after the operation. 14% had reduced EQ-5D _index_ scores—by more than 0.1—one year after the operation, and one third had less changes (less than 0.1) in their EQ-5D _index_ scores.

This first attempt to collect a whole sample of orthopedic conditions makes it possible to perform cost-utility analysis. A QALY is defined as 1 year in full health. Estimation of QALYs requires data on survival and the corresponding health state score, the health status reflecting the HRQOL of the individual, on a scale from 0 (dead) to 1 (full health) (Gold et al. 1996, Meunning and Gold 2001, Drummond et al. 2005). If utilities are multiplied by the amount of time spent in that particular health state, then they become QALYs. QALYs allow for varying times spent in different states by calculating an overall score for each patient. For the studies in which the follow-up is 1 year, the mean change in utility scores over the 1-year period can be directly interpreted as the MID for a QALY. QALYs may have the potential to influence public policy and decisions about resource allocation.

If baseline characteristics are controlled for the EQ-5D data, our findings could be used for comparison between hospitals. Comparison between provider units in different hospitals or between consultant specialities within a single institution can provide important information that might be applied for benchmarking or performance management.

In addition to clinical priority assessment, criteria in elective orthopedic surgery EQ-5D could be used (NKO 2009). Patients with low scores have low autonomy and should be given high priority (Government 2003, NKO 2009). We found that one third of all patients had a low preoperative HRQOL according to EQ-5D _index_ score and two-thirds of them improved considerably. In future, “soft” HRQOL data (e.g. EQ-5D) might be included in the preoperative evaluation as well as more old-fashioned “hard” data such as radiology. However, to use the instrument in order to make priorities between groups of orthopedic surgical procedures seems to be more controversial, as the patients' individual EQ-5D _index_ scores differed substantially.

The present study has several limitations. It is a prospective follow-up study of patients who underwent surgery, not a prospective randomized controlled trial comparing surgery to nonoperative treatment. However, most of the surgery performed involved accepted interventions (NKO 2009). At baseline, we lost 15% of all patients scheduled for elective surgery. If not all patients are reached at baseline, the patients with the most severe symptoms could be left out and the results would be biased towards patients with less symptoms. However, the numbers of patients included and the response rates were high, apart from for the group of patients with diabetes/infection. For patients who were operated on for diabetes/infection, our results may therefore have been underestimated.

The department only mailed 1 follow-up questionnaire to the patients and no reminders, which led to a loss of more than 40% of those initially included in the study. In the dropout analysis, no major difference was found in the preoperative EQ-5D _index_ scores between the responders and the non-responders. However, the responders tended to be women and to be older, causing our results to be a conservative interpretation. In this analysis, no information on patient co-morbidities or on other types of interfering conditions was collected. Thus, the study can be considered to represent a cross-section of orthopedic patients who undergo surgery at a university hospital.

We selected 1 year as a time outcome measurement because it was an easy endpoint. In some groups of patients (e.g. elderly), it might have been better to have had a shorter time frame because many other factors may have impaired the results.

The choice of algorithm used to convert self-classification scores can affect the index-based score, as shown in a study that compared UK and US scoring algorithms in patients undergoing percutaneous coronary intervention (PCI) (Shrive et al. 2007). However, while country-specific societal preferences may reduce the scope in comparing HRQoL estimates across studies from different countries, they are more helpful for local decision-making, especially when allocating resources within national healthcare programs.

The EQ-5D instrument has potential limitations. It may lack responsiveness to small but clinically important changes in health (Dawson et al. 2001). In the subgroups of patients who were operated on for elbow/hand, shoulder, and foot problems, we noted only minor health changes. The lack of minimal important differences (MID) for this group must be considered. It is also important to add condition-specific instruments in evaluating outcome after orthopedic surgery.

The bimodal distribution of EQ-5D scores that we found preoperatively and at the 12-month follow-up has also been reported by others (Conner-Spady et al. 2001, Xie et al. 2007). The EQ-5D algorithm tends to cluster scores in the upper extremity close to 1.0, and around 0.45. We strongly believe that it is the structure of the instrument that causes this phenomenon rather than the fact that it appears to highlight 2 subgroups of patients. This has also been noted in other studies (Rivero-Arias et al. 2005, Jansson et al. 2009).

We consider that our cohort represents patients in general who have undergone orthopedic surgery. This is the largest orthopedic cohort to be studied regarding HRQL so far, with 426 diagnoses and 446 orthopedic procedures. It could be questioned why we divided the cohort into 15 groups according to anatomical region and type of surgery, but it would have been difficult to present the results in any other way due to the large number of procedures. The drawback of this is that we lost the possibility of presenting details of specific diagnoses and procedures. We notice that our large cohort had a low HRQOL according to EQ-5D index score. A major strength in our report is that we matched our cohort with the Swedish EQ-5D reference population survey (Burström et al. 2001, 2003). We compared all patients older than 20 years of age and in spite of the finding that most patients felt an improved quality of life, the average preoperative EQ-5D index score of 0.54 is among the lowest reported in the literature so far. In the population survey (Burström et al. 2001) it was found that patients with low back pain scored 0.55, patients with stroke 0.43 and those with depression 0.38.
